# Synthesis and Biological Evaluation of a Novel Glucosylated Derivative of Gadolinium Diethylenetriaminepentaacetic Acid for Tumor Magnetic Resonance Imaging

**Published:** 2019

**Authors:** Massoud Amanlou, Elham Hashemi, Mohammad Ali Oghabian, Mehdi Shafiee Ardestani

**Affiliations:** a *Department of Medicinal Chemistry, Faculty of Pharmacy and Pharmaceutical Sciences Research Center, Tehran University of Medical Sciences, Tehran, Iran. *; b *Medical Physics Department, Faculty of Medicine, Tehran University of Medical Sciences, Tehran,Iran.*; c *Department of Radiopharmacy, Faculty of Pharmacy, Tehran University of Medical Sciences, Tehran, Iran.*

**Keywords:** Cancer diagnosis, Contrast agent, Gd^3+^-1-(4-isothiocyanatobenzyl) diethylene tri amine penta acetic acid, Magnevist, MRI

## Abstract

Cancer detection in early stage using a powerful and noninvasive tool is of high global interest. In this experiment, a small-molecular-weight glucose based derivative of Gd^3+^-1-(4-isothiocyanatobenzyl) diethylene tri amine penta acetic acid (Gd^3+^-p-SCN-Bn-DTPA-DG) as a novel potential MR imaging contrast agents was synthesized. Gd^3+^-p-SCN-Bn-DTPA-DG was synthesized with reacting of Glucosamine and 1-(4-isothiocyanatobenzyl) diethylene triamine penta acetic acid then loaded by gadolinium to make novel agent of functional MR imaging. The relaxivity, *T*_1_, *T*_2_ relaxation times, and cell toxicity of this contrast agent were studied. The results demonstrated that the sugar moieties linked to Gd^3+^-p-SCN-Bn-DTPA efficiently increase its cellular uptake in normal cells 25% and in cancereous cells upto 67%. The Gd^3+^-p-SCN-Bn-DTPA-DG significantly (*p* < 0.05) decreased MCF-7 tumor cell numbers without any significant toxicity on normal human kidney cells. Finally, it displayed an intense signal on *T*_1_ weighted with respect to the unlabeled cells. Based on the findings from the present research Gd^3+^-p-SCN-Bn-DTPA-DG be a potential breast molecular imaging. However, further investigations by anticancer studies are in the pipeline.

## Introduction

The ability to detect tumors at an early molecular stage would be a most important step toward the goal of suffering from the disease. The development of tumor-targeted delivery systems has opened the potential for delivery of imaging agents. (1) 

Magnetic resonance imaging (MRI) is one of the most employed non-invasive diagnostic assisted imaging techniques because of its liability to provide sensitive and marginal anatomical data in the early diagnosis of malignancy. (2-4) Contrast agents are vastly employed to enhance the imaging quality in MRI. (5, 6) These contrast substances contain paramagnetic metals like gadolinium. Gadolinium is a rare earth element. (7) It shows paramagnetic properties because its ion has seven unpaired electrons. 

Whereas free gadolinium is extremely toxic and needs to be controlled performing a variety of linear or macrocyclic metal-chelates. Chelation reduces the prospected gadolinium toxicity. (8, 9) 

Despite development in the synthesis of contrast agents, large numbers are yet restricted by low specificity (10). One of the most common paramagnetic contrast agents used in cancer diagnostics is Magnevist but it cannot cross the cell membranes and it is rapidly excreted in the urine. (9) The presence of the ligand/antibody on the linear and macrocyclic chelates facilitates the entry of the chelates into the cells through binding of the targeting molecule by its receptor followed by internalization of the bound them via receptor-facilated endocytosis, a highly effective cell entry pathway. (11, 12) This modification of chelates results in their being able to not only selectively deliver it to tumor cells. (13-14)

Targeting molecule that can be a ligand, such as folate (15), aptamers (16), carbohydrate (17-19), an antibody or an antibody fragment (20-21), directed against a cell surface receptor. Glucose is excellent tumor-detection agents (22-24). It has high cellular uptake due to over expressed glucose transporters (GLUTs) in cancer cells. (25) Glucose analogue is an excellent tumor-diagnosis agent whose uptake level correlates with tumor proliferation. 2-fluoro-2-deoxy-D-glucose molecule (^18^FDG) is a very successful positron imaging radiopharmaceutical of tumors such in Positron Emission Tomography (PET). (26-27) A systemically tumor-targeting delivery system has been developed in our laboratory for use in cell imaging. (23-24) These nanocarriers are composed of a dendrimer (23) or mesoporous silica nanospheres (MSN) (24) for detection cancer cells. Surface functionalized MSNs or Dendrimers were also used for selectively targeting cancer cells using cancer specific targeting molecules. (23- 26)

In this study, glucose derivative of Gd^3+^-1-(4-isothiocyanatobenzyl) diethylenetriamine penta acetic, as an alternative to ^18^FDG, was synthesized and characterized *in-vitro* with the goal of the high intracellular imaging potential. 

*Materials and Methods*

*Material*

1-(4-isothiocyanatobenzyl) diethylene tri amine pentaacetic was purchase from Macrocylclics USA. The GdCl_3_·6H_2_O (99%) was purchased from Sigma Aldrich (USA), and used without any further modifications. Dialysis bag covering 500-1000 D cut off was provided from the spectrum Comp. (USA). Fetal bovine serum (FBS; Invitrogen, Beijing, China) and penicillin – streptomycin were also obtained from sigma. Other materials were provided from Merck and Sigma companies. 

Human Breast cancer cells (MCF-7) were provided from the National Cell Bank of Pasteur Institute, Iran. MCF-7 cell line were subsequently cultured in Dulbecco’s modified Eagle’s medium (DMEN) supplemented with 5% fetal bovine serum (without heat-inactivation), and with inclusion of 1% penicillin – streptomycin and incubation at 37 °C and 5% CO_2_. 

*Instrumentation*

The Gadolinium was assessed by using inductively coupled plasma atomic emission spectrometry (ICP-AES, Optima 2300, Perkin-Elmer, and Boston, MA, USA). Fourier transform infrared spectra were obtained by an Equinox 55 spectrophotometer (Bruker, Ettlingen, Germany). Magnetic resonance imaging (MRI) was carried out on a 1.5 Tesla scanner (Siemens, Erlangen, Germany).

Absorbance was observed at 450 nm using an ELX800 absorbance microplate reader (Bio-Tek Instruments Inc, Winooski, VT, USA). ^1^HNMR spectrums were studied on a Bruker AMX-300 spectrometer (solvent: deuterium oxide, pD _ 9 or CDCl_3_).

LC-MASS was obtained on an Agilent Technologies Inc. (NYSE: A).

*Synthesis Glucosylated Derivatives of 1-(4-isothiocyanatobenzyl) diethylene tri amine pentaacetic (Gd* ^3+^ *-p-SCN-Bn-DTPA-DG)*

100 mg D-glucosamine hydrochloride was gently neutralized using excess quantities of sodium bicarbonate (9). The reaction was allowed to stir for at least 30 min and filtered. The excess quantity of ascorbic acid (200 mg) was thereafter drop wised to the solution. 

**Figure 1:**
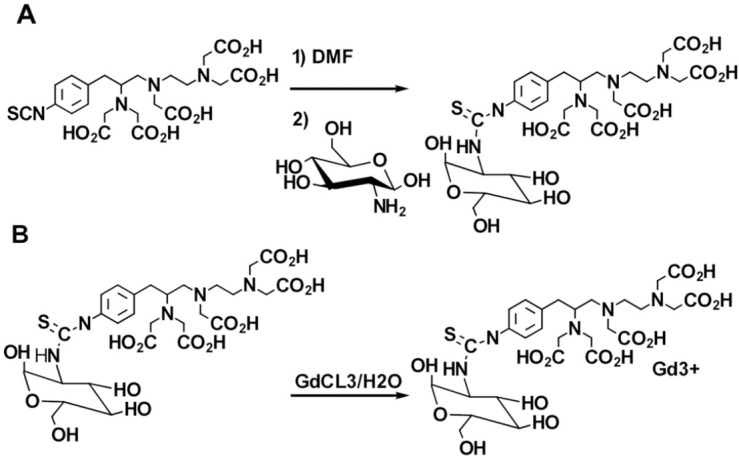
Schematic of synthesis of a) p-SCN-Bn-DTPA-DG b) Gd3+-p-SCN-Bn-DTPA-DG

The reaction was rapidly lyophilized, and a mild yellowish powder was yielded, 98% (see Figure 1). p-SCN-Bn-DTPA (0.1 mmol) was dissolved in distilled water (10 mL), and then D-deoxy-glucosamine (DG) 0.333 mmol was drop wised. The reaction solution was allowed to be stirred for 30 min. Thin-layer chromatography demonstrated only one spot regarding the final product and no evidence of the starting material. The p-SCN-Bn-DTPA -DG was purified using a dialysis bag with a cutoff point of 500 Da in water for a course of one day. The obtained solution was subjected to lyophilize. p-SCN-Bn-DTPA -DG as a white powder was obtained with an overall yield of 98%.

The p-SCN-Bn-DTPA -DG (1 mmol) was reacted in a medium containing water and GdCl_3_ (1 mmol) at RT for at least 60 min. The reaction mixture was then dialyzed against the double distilled water employing dialysis bag (Figure 1).Yield: 89%. 

^1^ *H NMR(500 MHz, DMSO)*

ppm: 1.230 (s, 4H, -OH), 1.672 (s, 2H, -CH), 2.307 (s, 1H, -CH- ), 2.683 (d, 1H, -CH), 2.997 (s, 4H, -CH_2_- ), 3.207 (s, 4H, -CH_2_- ), 3.503 (m, 10H, -CH_2_- ), 4.170 (m, 2H ), 4.486 (d, 1H, -CH- ), 4.960 (s, 1H, -CH- ), 7.043 (d, 2H, Ar-CH- ), 7.188 (d, 2H, Ar-CH- ), 8.481 (s, 2H, -NH-) , 10.406 (s, 5H, -COOH ) .

LC-Mass (for p-Bn-SCN-DTPA-DG): M^+^ (828.2000), M^+^-(–COOH) (784.2000), M^+^ - 2 (–COOH) (740.3000), M^+^ - 3 (–COOH) (693.3000) LC-Mass (for Gd^3+^-p-Bn-SCN-DTPA-DG): M^+^ (985). 

*Cell viability (MTT) assay*

The MCF-7 and HEK 293 cell lines were grown in 96-ELISA well plates (5 × 10^5^ cells per well), which each well was subjected to addition of 200 μL of Dulbecco’s modified Eagle’s medium and 10% fetal bovine serum. After enough culture medium for 24 h, the medium was then excluded and exchanged with Dulbecco’s modified Eagle’s medium supplemented with 1% fetal bovine serum in the absence or presence of five concentrations Gd^3+^-p-SCN-Bn-DTPA-DG (100 nM, 200 nM, 400 nM, 600 nM, 800 nM ) and then incubated for at least 48 h at 37 °C. MTT aqueous solution (20 μL of 5 mg/mL) was incorporated to each well and afterwards incubated at 37 °C for 4 h in 5% CO_2_; cellular reducing of MTT by mitochondrial dehydrogenase enzyme in vital cancerous cells composites a blue formazan product, which can be estimated quantitatively by a microplate Elisa reader apparatus at 570 nm. (23-24)

*Cellular uptake assay*

To assess the intracellular uptake of Gd^3+^-p-SCN-Bn-DTPA-DG, the subjected cells were dispersed into six-well plates considering a concentration of 2 × 10^5^ cells per each well and then incubated at 37 °C / 5% CO_2_ for at least 24 h. Gd^3+^-p-SCN-Bn-DTPA-DG (400 nM), Magnevist (400 nM) was exposed to the wells, which contained 1 mL of medium. The cells were allowed to be incubated at 37 °C with 5% CO2 for at least 90 min. By employing 500 μL of phosphate-buffered saline (PBS) the cells were then washed twice and then centrifuged at 1500 rpm for 10 min and reconstituted with 100 μL of PBS. Finally, total amounts of Gd^3+^ cellular uptake was definitely obtained by ICP-AES instrumentation. 

*Statistical analysis*

Data means comparisons were calculated by one-way analysis of variance (ANOVA) performance. In addition, the analyzed data were depicted as Mean ± SEM and P < 0.05 was elected as statistical significant concept.

*Measurements on MRI*

Relaxation times measurements for Gd^3+^-p-SCN-Bn-DTPA-DG were calculated based on the previous studies (9, 24) at differ­ent concentrations of 0.1612, 0.1075, 0.0537, and 0.0268 mM. Different spin echo as well as gradient echo protocols were employed, with a 1.5 Tesla MRI equipped with a head coil. A rapid protocol was used to determine the T_1_ maps. Standard spin echo was respectively as follows: echoes 1; TE 15 msec; TR 50, 100, 200, 400, 600, 1000, and 2000, 5000 msec; matrix 512*384; slice thickness 4 mm; field of view 25 cm; NEX 3; and pixel bandwidth 130. Multiple spin echo protocols were also performed for T_2_ measurement. Standard spin echo particulars were respectively as follows: echoes 4; TE 13.2, 26.4, 92.4, 105.6 , 118.8, 132.0, 145.2,158.4, 224.4, 250.8,264.0,303.6, 316.8,356.3,396.0, 422.0 msec; TR 3000 msec; matrix 512*384; slice thickness 4 mm; field of view 25 cm; and NEX 3. For quan­titative data analysis, the obtained MRI images were transferred to DICOM Works software version 1.3.5 (Digital Imaging and Communications in Medicine, Rosslyn, VA, USA). (9, 24) 

*Theory/calculation* 

The current experiments, for the first, explore a simple synthetic way to synthesize and *in vitro* biologically evaluation of novel Gd^3+^-p-Bn-SCN-DTPA-DG conjugate as a very successful MR Molecular imaging agent. In future studies, for further assessment regarding the conjugate liability, *in-vivo* experiments including animal or clinical would be desirable to be performed. 

## Results

*Cell viability assay*

**Figure 2:**
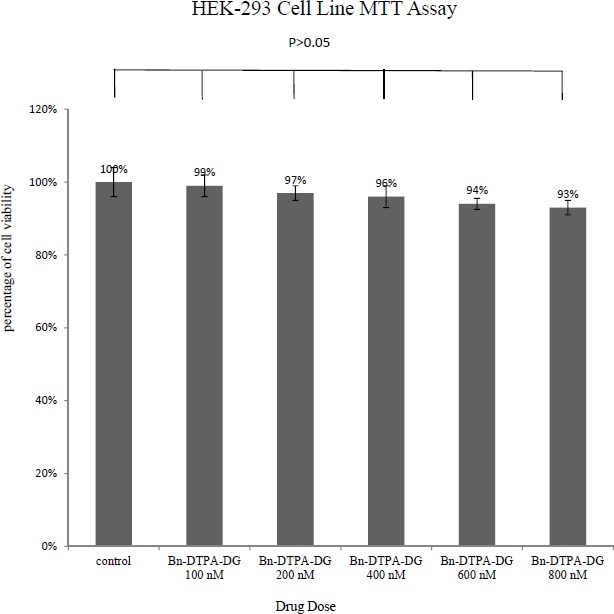
MTT results of 48 h of Bn-DTPA-DG exposure to the HEK 293 cell line

Notes: The in vitro cytotoxicity of Bn-DTPA-DG was examined at five different concentrations. Each concentration was performed in triplicate and the mean ± standard deviation was shown. Bn-DTPA-DG -labeled cells had insignificant differences in cell viability at these concentrations (P > 0.05).

**Figure 3:**
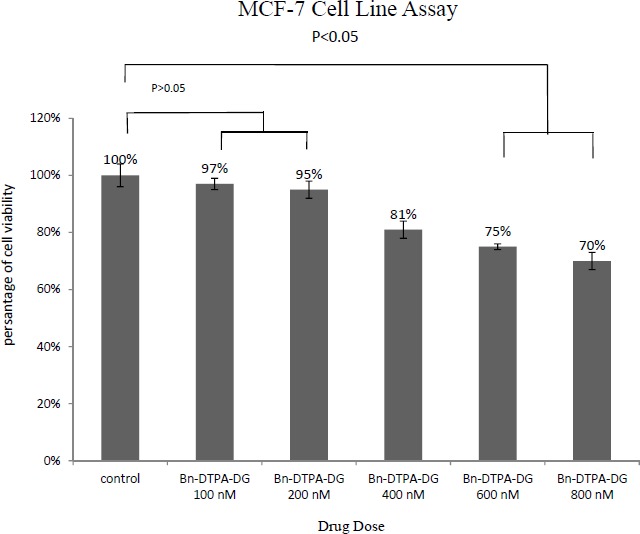
MTT results of 48 h of Bn-DTPA-DG exposure to the MCF-7 cell line

Notes: The in vitro cytotoxicity of Bn-DTPA-DG was examined at five different concentrations. Each concentration was performed in triplicate and the mean ± standard deviation was shown. Bn-DTPA-DG -labeled cells had insignificant differences in cell viability at 100 nM and 200 nM and had significant differences in cell viability at 400 nM, 600 nM, 800 nM (P > 0.05).

MTT assays were respectively performed employing two different cancer and normal cell lines, the MCF-7 and HEK 293 to determine whether Bn-DTPA-DG complying a cytotoxic liability. Figure 2. and Figure 3. show a comparison of the MCF-7 and HEK 293 cell lines while incubated for at least 48 h at diverse dosages of Bn-DTPA-DG. 

The Bn-DTPA-DG dosages were complied with that of the control (0 μg/mL), demostrating that cellular viability was not significantly affected at the concen­tration range analysis.

*Gadolinium Cellular Assay*

The total cellular amounts of Gd^3+^-p-SCN-Bn-DTPA-DG and Magnevist for MCF-7 and HEK 293 cell lines were determined, as shown in Figure 6 and Figure 7. The mass spectroscopic results demonstrate that cellular uptake Bn-DTPA-DG was about 6.6 times more than Magnevist for the HEK 293 cell line and 14 times more than Magnevist for the MCF-7 cell line. The analysis confirms the potential role of glucose in Gd^3+^-p-SCN-Bn-DTPA-DG.

*Relaxivity Assay*

The MRI relaxation times for Gd^3+^-p-SCN-Bn-DTPA-DG were estimated employing a 1.5 Tesla MRI scanner (Figure 6) (Figure 7). The Gd^3+^-p-SCN-Bn-DTPA-DG demonstrated large longitudinal (*r*_1_) and transverse (*r*_2_) relaxivities. The *r*_1_ and *r*_2_ values were 13.03 mM^−1^s^−1^ and 31.24 mM^−1^s^−1^, respectively and the *r*_2_*:r*_1_ ratio was 2.3 (Figure 6) (Figure 7). The presenting findings are significantly comparable with that of standard drug Gd^3+^-DTPA-known also as Magnevist^®^ (3.36 mM^−1^s^−1^ in distilled water). 

## Discussion

Cancer cells intake more glucose (as cell energy supplier) than normal cells to exceed their growth and to compensate their insufficient glucose intake. Glucose analogue is an excellent tumor-diagnosis agent whose uptake level correlates with tumor proliferation and through upregulation of specific transporters (GLUTs).

In the present study, for the first, Gd^3+^-p-SCN-Bn-DTPA-DG was synthesized with reacting of Glucosamine and 1-(4-isothiocyanatobenzyl) diethylene tri amine penta acetic acid then loaded by gadolinium to make novel agent of functional MR imaging.

While Magnevist does not cross the cell membranes, cellular uptake of Magnevist has been observed using DG-conjugated Gd^3+^-p-SCN-Bn-DTPA. 

Intra-cellular uptakes of Gd^3+^-p-SCN-Bn-DTPA-DG and Magnevist on MCF-7 and HEK 293 cell lines were measured using a special kind of mass spectroscopy as stated. The data analysis indicated that the Magnevist intracellular uptake was 3.78% and 4.76% on HEK 293 and MCF-7, respectively. Outcome indicated that Magnevist does not enter into the cells appropriately. Additionally, the intracellular uptake of Gd^3+^-p-SCN-Bn-DTPA-DG was 25.07% and 67.18% on HEK 293 and MCF-7, respectively. Result indicated that cellular uptake of Gd^3+^-p-SCN-Bn-DTPA-DG on MCF-7 was about 2.67 times more than on HEK 293. These results attributed to over expression of GLUTs in cancer cells and Gd^3+^-p-SCN-Bn-DTPA-DG is entered inside the viable cells specifically malignancies by special kind of glucose carriers (glut family). Other researches confirm these results. For example cellular uptake of glycosylated Gd^3+^- base Mesoporous silica nanospheres on HT 29 cell line was 75.61%. In the other research (9, 23, 24, 27-29).

The results showed that MCF-7 could also be reliably labeled with Gd^3+^-p-SCN-Bn-DTPA-DG, without using transfection agent. This property might be sufficiently employed for intracellular uptake quantifications. Current observations depicted that the significant Gd^3+^ internalization obtained through a receptor-mediated endocytosis mechanism. The description on the situation is that upon binding of the conjugated metal containing-sugar, the transporting carrier is unable to keep on with the successive stages that bring sugar into the cytoplasm. Consequently, it comes to the clathrine-rich space to be trapped in endosomal vesicle medium. 

The cytotoxicity studies have indicated that Gd^3+^-p-SCN-Bn-DTPA-DG labeled cells exhibited in significant toxcicity on HEK 293 as compared to unlabeled controls but this contrast agent showed significant toxicity on MCF-7 cell line with increased in the concentration (Figure 5) The results suggested that 100 μgmL^-1^ Gd^3+^-p-SCN-Bn-DTPA-DG is suggesting the optimum dosage to be employed for cell-labeling and imaging.

**Figure 4:**
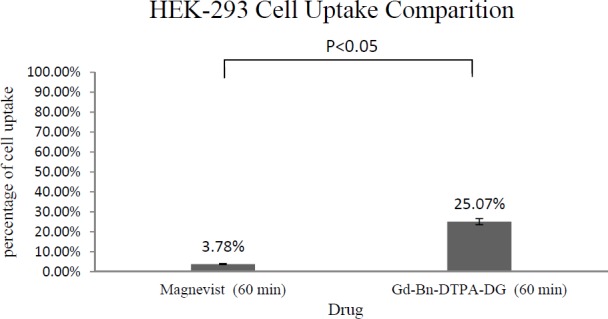
Cell uptake assay of Gd^3+^-Bn-DTPA-DG and Magnevist on HEK 293: Result was indicated of glucose effect on intracellular uptake (P < 0.05).

**Figure 5:**
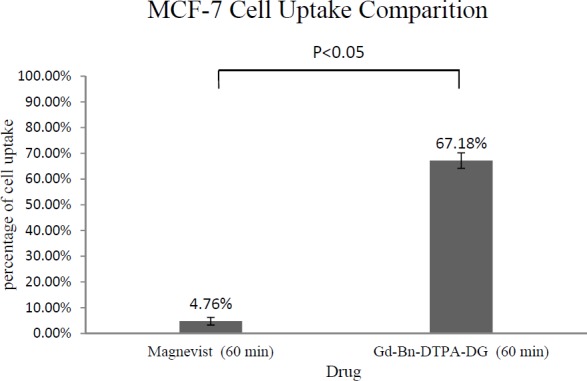
Cell uptake assay of Gd^3+^-Bn-DTPA-DG and Magnevist on MCF-7: Result was indicated of glucose effect on intracellular uptake (P < 0.05)

**Figure 6:**
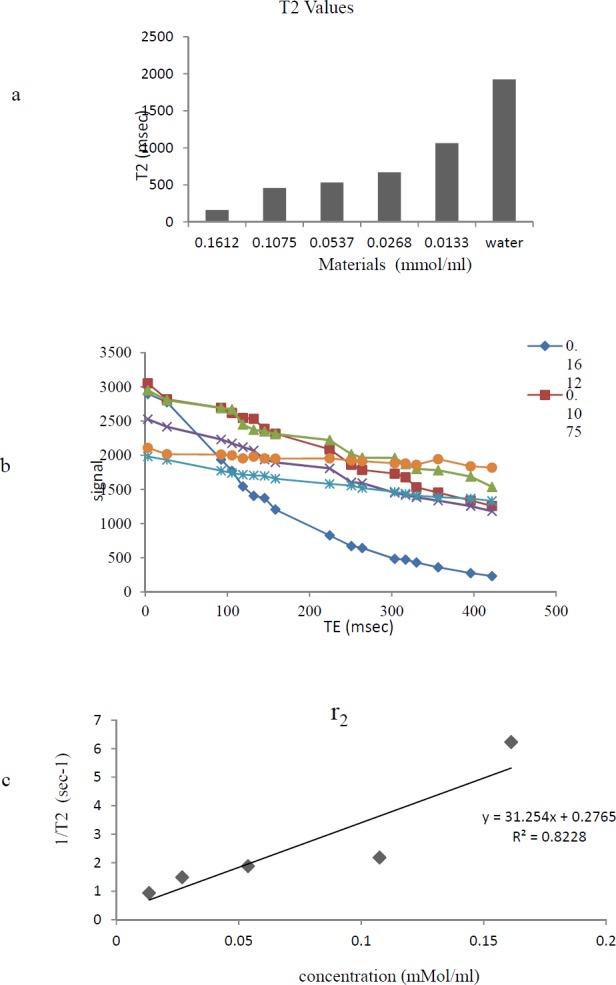
a) Effect of Gd^3+^-Bn-DTPA-DG on T2 relaxation times to a significantly greater extent than water;b) T_2_ data based on spin echo and gradient echo protocols;. C) The r_2_ relaxivity curves of Gd^3+^-Bn-DTPA-DG

**Figure 7:**
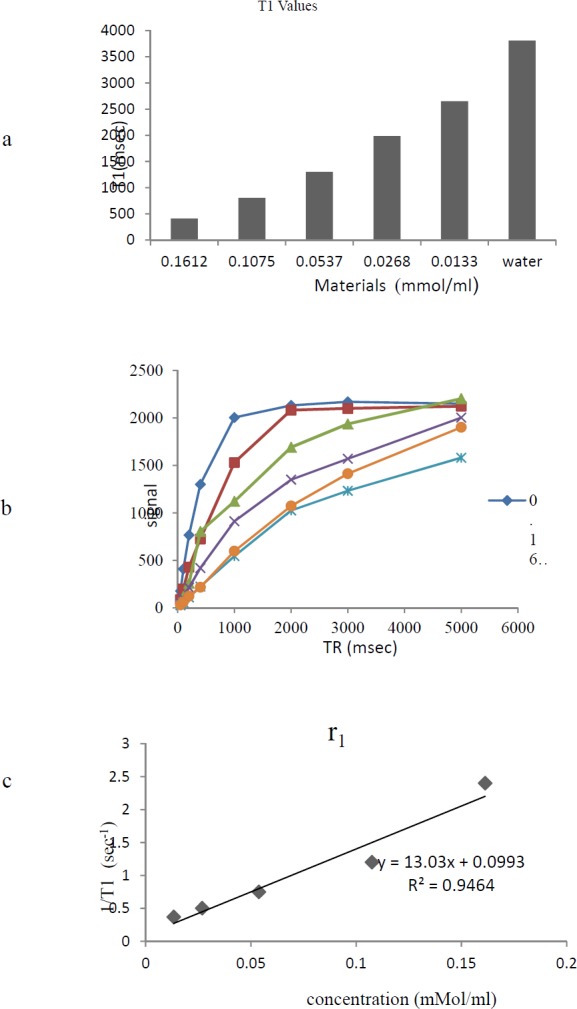
a) Effect of Gd^3+^-Bn-DTPA-DG on T_1_ relaxation times to a significantly greater extent than Water;b) T1 data based on spin echo and gradient echo protocols. C) The r_1_ relaxivity curves of Gd^3+^-Bn-DTPA-DG

**Figure:**
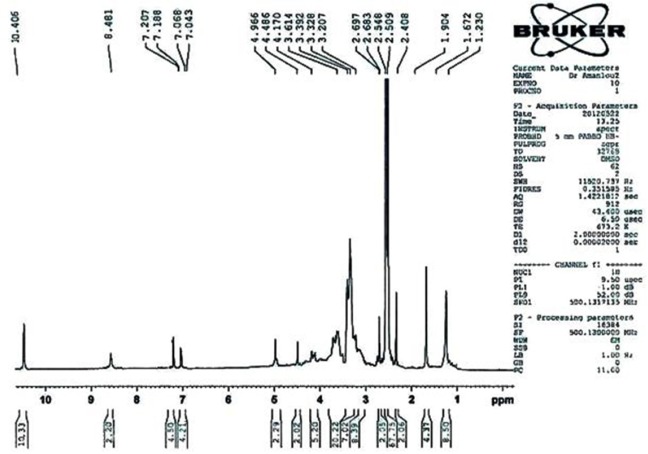
1H NMR(500 MHz, DMSO)

ppm: 1.230 (s, 4H, -OH), 1.672 (s, 2H, -CH-), 2.307 (s, 1H, -CH-), 2.683 (d, 1H, -CH-), 2.997 (s, 4H, -CH2-), 3.207 (s, 4H, -CH2-), 3.392-3.614 (m, 10H, -CH2-), 4.170 (m, 2H), 4.486 (d, 1H, -CH-), 4.960 (s, 1H, -CH-), 7.043 (d, 2H, Ar-CH-), 7.188 (d, 2H, Ar-CH- ), 8.481 (s, 1H, -CO-NH-), 10.406 (s, 5H, -COOH).

**Figure:**
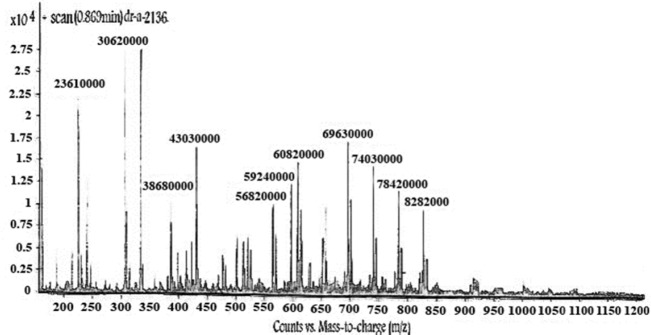
LC-Mass: M^+^828.2000(), M^+^)- –COOH784.2000((), M^+^ - 2(–COOH) (740.3000), M^+^ - 3(–COOH) (693.3000).

The relaxivity studies have shown that Gd^3+^-p-SCN-Bn-DTPA-DG labeled cells *r*_1_ values *was* 13.03 mM^−1^s^−1 ^(Figure 8). The *T*_1_-weighted image data were regarded to the decrease of *T*_1_ relaxation times (Figure 7.).

Relaxivities were also assessed, and the *r*_2_*:r*_1_ ratio obtained 1.3, demonstrating that Gd^3+^-p-SCN-Bn-DTPA –DG was also a potent T_1_-weighted contrast media imaging agent. Also, Gd^3+^-p-SCN-Bn-DTPA-DG able to decrease *T*_1_*/T*_2_ relaxation times to a significantly greater amount comparing to water (Figure 6-7).

In summary, Gd^3+^-p-SCN-Bn-DTPA-DG depicts several positive states; its minimal size permits its rapid diffusion into the tissue to obtain the cancer targets. The proposed gadolinium agent internalized into the cells by receptor mediated endocytosis, therefore, preventing undesirable interaction with the other molecular and cellular events. Our data evidenced that Gd^3+^-p-SCN-Bn-DTPA-DG could be good candidates as cancer cell imaging. Incorporation of D-glucose or D-glucosamine to the currently available extracellular contrast imaging agent Gd^3+^-DTPA may subsequently significantly increase its Cellular uptake liability.

## Conclusions

As expected, glucose conjugated to the Gd^3+^-p-SCN-Bn-DTPA. The covalent bond formed between the p-SCN-Bn-DTPA and glucosamine is resistant to any biological *in-vivo* disruption. Besides, p-SCN-Bn-DTPA-DG can be easily/ efficiently labeled with Gd^3+ ^ions. No significant toxicological features *in-vitro* was observed for HEK 293, and these are further important advantages of Gd^3+^-p-SCN-Bn-DTPA.

There is a significant similarity between the cellular uptake of Gd^3+^-p-SCN-Bn-DTPA and ^18^FDG in tumors. Our results point to the potential use of Gd^3+^-p-SCN-Bn-DTPA conjugates as functional MRI contrast agents. As a result of observed confirmations from the present research, Gd^3+^-p-SCN-Bn-DTPA conjugate is a potential selective viable tumor molecular imaging agent and seems to be further clinically studied in the near future. 
